# H_2_ Is a Major Intermediate in *Desulfovibrio vulgaris* Corrosion of Iron

**DOI:** 10.1128/mbio.00076-23

**Published:** 2023-02-14

**Authors:** Trevor L. Woodard, Toshiyuki Ueki, Derek R. Lovley

**Affiliations:** a Department of Microbiology, University of Massachusetts—Amherst, Amherst, Massachusetts, USA; b Institute for Applied Life Sciences, University of Massachusetts—Amherst, Amherst, Massachusetts, USA; University of Tennessee at Knoxville

**Keywords:** *Desulfovibrio vulgaris*, biocorrosion, extracellular electron transfer, iron, sulfate reduction

## Abstract

Desulfovibrio vulgaris has been a primary pure culture sulfate reducer for developing microbial corrosion concepts. Multiple mechanisms for how it accepts electrons from Fe^0^ have been proposed. We investigated Fe^0^ oxidation with a mutant of *D. vulgaris* in which hydrogenase genes were deleted. The hydrogenase mutant grew as well as the parental strain with lactate as the electron donor, but unlike the parental strain, it was not able to grow on H_2_. The parental strain reduced sulfate with Fe^0^ as the sole electron donor, but the hydrogenase mutant did not. H_2_ accumulated over time in Fe^0^ cultures of the hydrogenase mutant and sterile controls but not in parental strain cultures. Sulfide stimulated H_2_ production in uninoculated controls apparently by both reacting with Fe^0^ to generate H_2_ and facilitating electron transfer from Fe^0^ to H^+^. Parental strain supernatants did not accelerate H_2_ production from Fe^0^, ruling out a role for extracellular hydrogenases. Previously proposed electron transfer between Fe^0^ and *D. vulgaris* via soluble electron shuttles was not evident. The hydrogenase mutant did not reduce sulfate in the presence of Fe^0^ and either riboflavin or anthraquinone-2,6-disulfonate, and these potential electron shuttles did not stimulate parental strain sulfate reduction with Fe^0^ as the electron donor. The results demonstrate that *D. vulgaris* primarily accepts electrons from Fe^0^ via H_2_ as an intermediary electron carrier. These findings clarify the interpretation of previous *D. vulgaris* corrosion studies and suggest that H_2_-mediated electron transfer is an important mechanism for iron corrosion under sulfate-reducing conditions.

## INTRODUCTION

Microbial corrosion of iron-containing metals is a substantial economic problem, but the mechanisms are poorly understood (1–3). Fundamentally, iron corrosion is the oxidation of metallic iron to ferrous iron:

(1) $${\text{Fe}}^{0}\to {\text{Fe}}^{+\text{2}}\;+\;{\text{2e}}^{-}$$

This reaction must be coupled with an electron accepting reaction to proceed. Thus, microorganisms can stimulate iron corrosion by either accelerating reaction 1 or by contributing to one or more reactions that consume the electrons from reaction 1.

**FIG 1:**
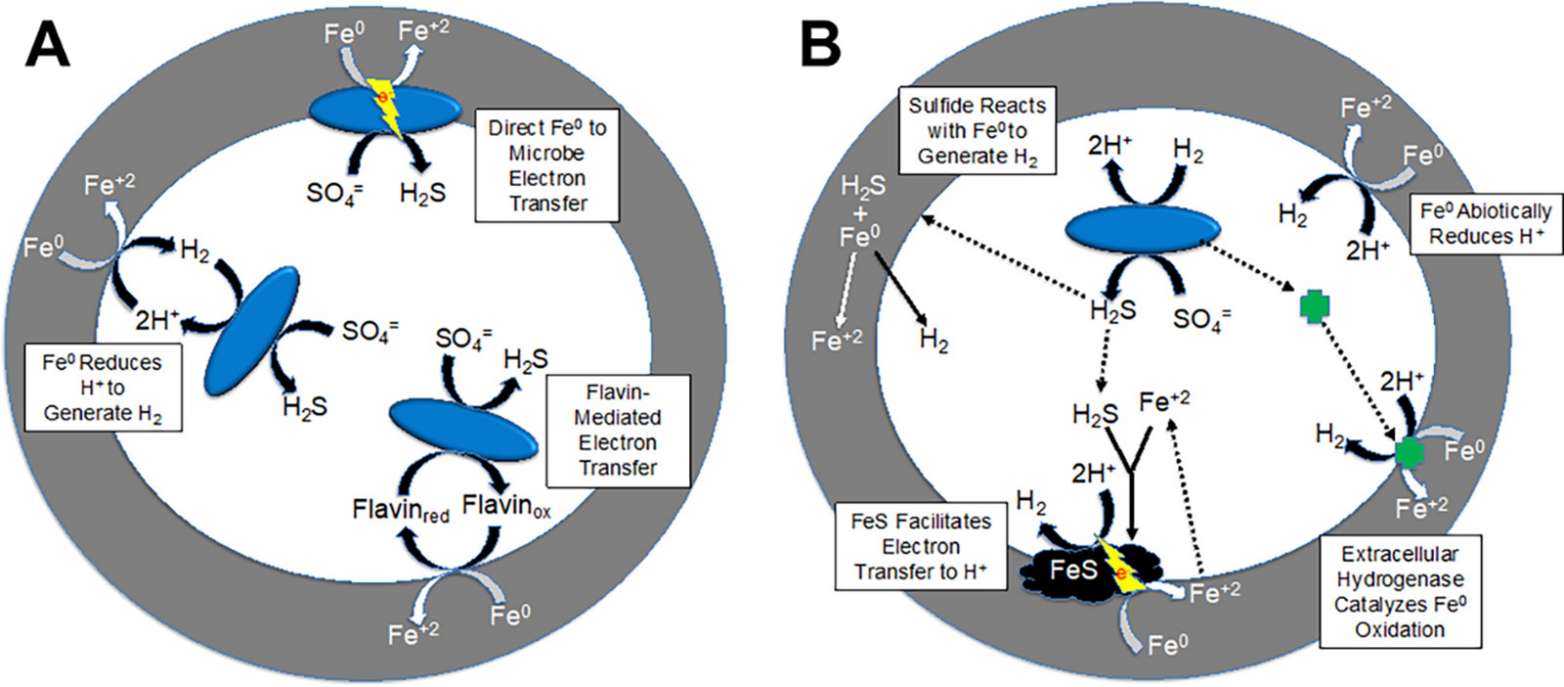
Previously proposed mechanisms for *Desulfovibrio* species to receive electrons during metallic iron corrosion. (A) The three primary mechanisms that have been proposed. (B) Diversity of potential routes for H_2_ generation.

Sulfate reducers are often implicated in iron corrosion (1, 4–6). *Desulfovibrio* spp. have been the most studied pure culture isolates for investigating iron corrosion under sulfate-reducing conditions, dating back to the some of the earliest studies on microbial corrosion (1, 3, 7). Three mechanisms for *Desulfovibrio* species to consume electrons derived from Fe^0^ oxidation have been proposed (Fig. 1A). The first mechanism proposed (8) was abiotic oxidation of Fe^0^ coupled to proton reduction to generate H_2_:

(2) $${\text{Fe}}^{0}\;+{\text{ 2H}}^{+}\to {\text{Fe}}^{+\text{2}}\;+{\text{ H}}_{\text{2}}$$

combined with consumption of the H_2_ via sulfate reduction:

(3) $${4\text{H}}_{\text{2}}\;+{\text{ SO}}_{\text{4}}{}^{=}\;+\;{2\text{H}}^{+}\to {\text{H}}_{2}{\text{S}}^{}\;+\;{4\text{H}}_{\text{2}}\text{O}$$

Several mechanisms that might enhance H_2_ production have been proposed (Fig. 1B). Hydrogenases released from moribund cells may accelerate reaction 1 by catalyzing H_2_ production from Fe^0^ (9). H_2_S generated from sulfate reduction may promote H_2_ production from Fe^0^ in two ways. Sulfide can react with Fe^0^ to generate H_2_ (1):

(4) $${\text{Fe}}^{0}\;+{\text{ H}}_{\text{2}}\text{S}\to \text{FeS }+{\text{ H}}_{\text{2}}$$

and iron sulfide precipitates might facilitate electron transfer from the Fe^0^ to H^+^, accelerating reaction 2 (2, 10).

One of the most intriguing proposed mechanisms for *Desulfovibrio* species to participate in iron corrosion is direct microbial electron uptake from Fe^0^ (1, 11–14). *D. ferrophilus* (previously known as strain IS*5*) can grow with H_2_ as the sole electron donor, but it was inferred to directly consume electrons from Fe^0^ based on the observation that it reduced sulfate faster than several other H_2_-oxidizing sulfate reducers (11). However, this inference relies on the unsubstantiated assumption that direct electron transfer is faster than H_2_-mediated electron transfer from Fe^0^ to microbes. Furthermore, possible adaptions in *D. ferrophilus* for enhanced growth on H_2_ derived from Fe^0^—such as producing an extracellular hydrogenase to accelerate Fe^0^ oxidation, a higher affinity for H_2_, or possibly a better capacity for attachment to Fe^0^—were not considered (3).

In subsequent studies, *D. ferrophilus* grew with pure Fe^0^ as the electron donor, but not with stainless steel (15). This distinction is important because pure Fe^0^ abiotically generates H_2_ via reaction 1 (16, 17), but stainless steel does not (18). In contrast to *D. ferrophilus*, stainless steel is an effective electron donor for *Geobacter* and *Methanosarcina* strains capable of direct electron uptake from Fe^0^ (15, 18, 19). Notably, protease digestion of *D. ferrophilus* extracellular proteins did not affect sulfate reduction rates with Fe^0^ as the electron donor (20), a result inconsistent with a microbe making direct electrical contact with Fe^0^ because protease degrades outer-surface electrical contacts (21). Therefore, the evidence available to date suggests that *D. ferrophilus* most likely accepts electrons from Fe^0^ via an H_2_ intermediate (15).

*D. vulgaris* is the most intensively studied sulfate reducer for biochemical and physiological investigations, and has served as a model sulfate reducer for many corrosion studies (7). Direct Fe^0^-to-microbe electron transfer has also been proposed for *D. vulgaris* (13, 14, 22), but as with the *D. ferrophilus* studies, the possibility of H_2_-mediated metal-to-microbe electron transfer was not rigorously eliminated. Studies with *Geobacter* (17, 18), *Shewanella* (23, 24), and *Methanosarcina* (19) species have provided evidence for direct electron uptake from Fe^0^ by (i) eliminating the possibility that H_2_ was serving as an electron shuttle between Fe^0^ and cells and (ii) demonstrating with gene deletions that outer-surface *c*-type cytochromes were required for electron uptake from Fe^0^. In contrast, no studies have previously been reported on *D. vulgaris* corrosion with strains that were unable to use H_2_ (7). *D. vulgaris* lacks outer-surface cytochromes (25), and no other *D. vulgaris* outer surface electrical contacts are known. Unlike the microbes previously shown to directly accept electrons from Fe^0^ (17–19, 23, 24), *D. vulgaris* does not directly reduce Fe(III) (26), an ability common to most microbes that can directly exchange electrons with extracellular electron donors and acceptors (27).

Higher rates of corrosion following the addition of riboflavin (14, 28, 29) led to the suggestion that riboflavin can function as an electron shuttle that Fe^0^ reduces:

(5) $${\text{Fe}}^{0}\;+{\text{ riboflavin}}_{\text{oxidized}}\;+\;{2\text{H}}^{+}\to {\text{Fe}}^{+2}\;+{\text{ riboflavin}}_{\text{reduced}}{\text{H}}_{2}$$

with *D. vulgaris* oxidizing the reduced riboflavin with the reduction of sulfate:

(6) $${\text{SO}}_{4}{}^{=}\;+\;{4\text{ riboflavin}}_{\text{reduced}}{\text{H}}_{2}\;+\;{2\text{H}}^{+}\to {\text{H}}_{2}\text{S }+\;{4\text{ riboflavin}}_{\text{oxidized}}\;+\;{4\text{H}}_{2}\text{O}$$

However, those studies did not determine whether Fe^0^ could donate electrons to riboflavin or whether reduced riboflavin can serve as an electron donor for sulfate reduction. The alternative possibility that riboflavin might stimulate other aspects of microbial metabolism was also not evaluated. Furthermore, electron transfer via H_2_ was still possible in those studies.

A rigorous strategy to evaluate the possibility of H_2_ serving as an intermediary electron carrier is to determine whether strains unable to use H_2_ as an electron donor can respire with Fe^0^ as the sole electron donor (17–19, 23, 30). In instances in which the wild-type strain of interest can consume H_2_, this can be accomplished by deleting genes necessary for H_2_ metabolism (17, 23, 30). A strain of *D. vulgaris* in which genes for all of the annotated hydrogenases on the genome were deleted is available as one of a large collection of mutant strains (31). We report here on studies on Fe^0^-dependent sulfate reduction conducted with this hydrogenase-deficient strain.

## RESULTS AND DISCUSSION

### Hydrogenase mutant unable to grow with H_2_ as electron donor.

**FIG 2:**
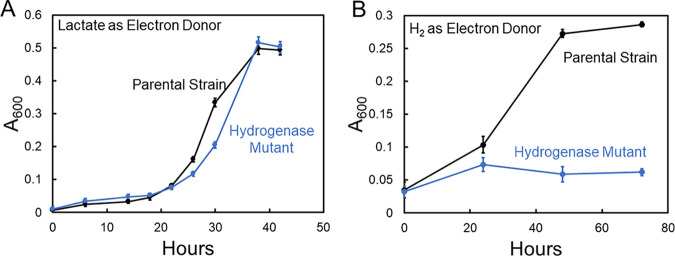
(A and B) Growth of D. vulgaris parental strain and hydrogenase mutant with sulfate as the electron acceptor and either lactate (A) or H_2_ (B) as the electron donor, as measured by culture turbidity. The data are presented as the means and standard deviations of triplicate incubations.

The hydrogenase-deficient mutant grew as well as the parental strain in medium with lactate as the electron donor and sulfate as the electron acceptor (Fig. 2A), but unlike the parental strain, the hydrogenase mutant did not grow in medium with H_2_ as the sole electron donor (Fig. 2B). These results suggested that the hydrogenase mutant was a suitable strain to evaluate the role of H_2_ as an intermediary electron carrier during growth with Fe^0^ as the electron donor.

### Hydrogenase mutant cannot reduce sulfate with Fe^0^ as electron donor.

**FIG 3:**
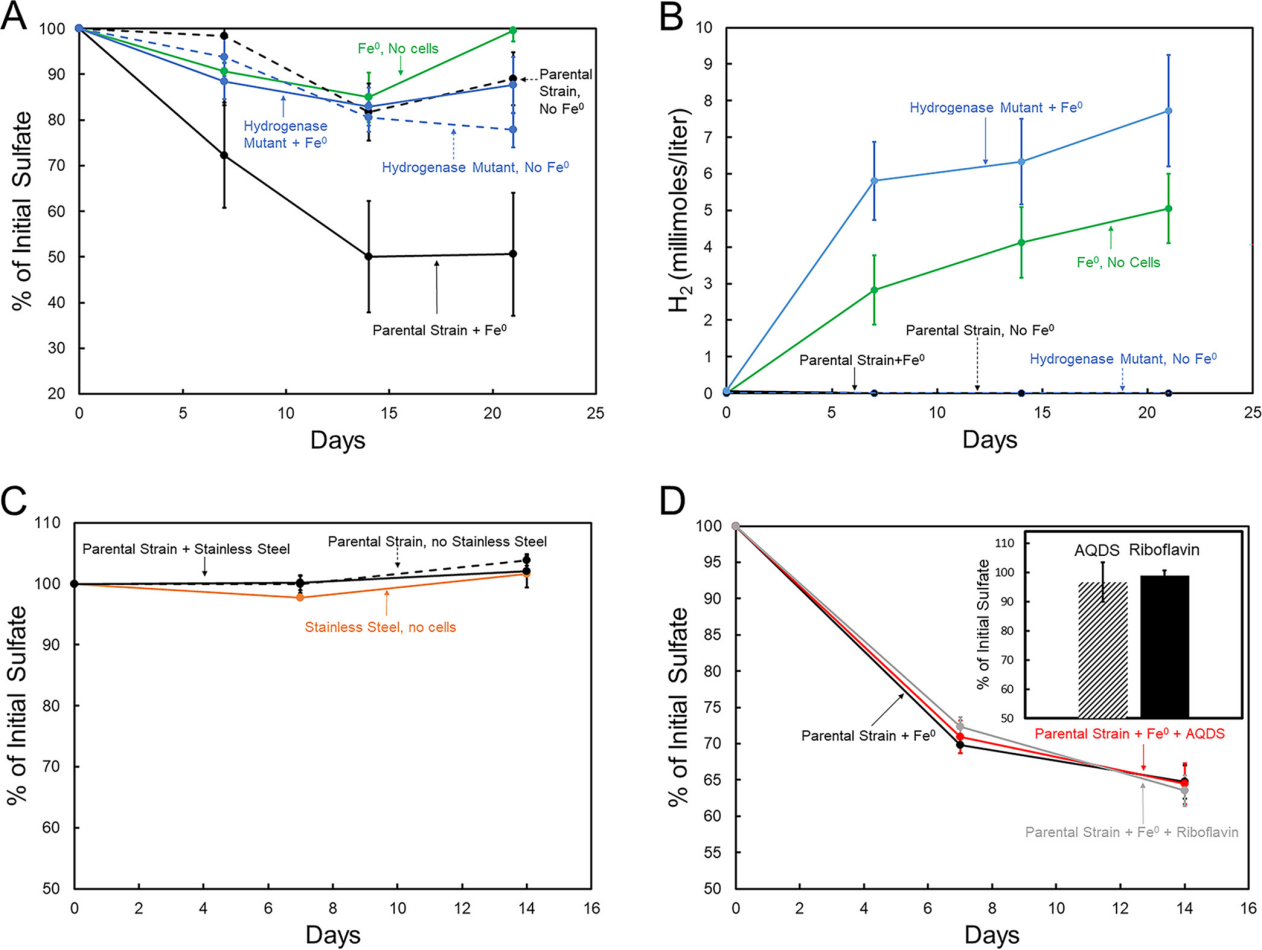
Sulfate reduction and H_2_ concentrations with iron as the electron donor in the presence of D. vulgaris parental strain or hydrogenase mutant. (A and B) Sulfate loss (A) or H_2_ concentrations (B) over time with pure Fe^0^ as the sole electron donor in the presence of the *D. vulgaris* strains and in controls without cells or without Fe^0^. (C) Lack of sulfate depletion with 316L stainless steel as the potential electron donor for the parental strain and in controls without cells or without stainless steel. (D) Sulfate loss over time in parental strain cultures amended with riboflavin, anthraquinone-2,6-disulfonate (AQDS), or no amendments, with Fe^0^ as the sole electron donor. The bar graph inset in panel D shows the lack of sulfate loss in cultures of the hydrogenase mutant with Fe^0^ as the electron donor and amendments of riboflavin or AQDS. The data represent the means and standard deviations of triplicate incubations.

The parental strain reduced sulfate with Fe^0^ as the sole electron donor, but the hydrogenase mutant did not (Fig. 3A). The slight decline in sulfate over time in cultures with the hydrogenase mutant could be attributed to carry over of lactate with the inoculum because the final sulfate levels for the hydrogenase mutant with Fe^0^ were the same as for the parental strain without Fe^0^ (Fig. 3A). As expected from previous studies under similar conditions (17), H_2_ accumulated in sterile controls (Fig. 3B), reflecting abiotic Fe^0^ oxidation coupled to H^+^ reduction. H_2_ also accumulated in cultures inoculated with the hydrogenase mutant, further demonstrating the inability of this strain to consume H_2_. H_2_ accumulated more in the hydrogenase mutant cultures than in the uninoculated control, probably due to the sulfide that was transferred along with the inoculum (see sulfide effect on H_2_ production below). In contrast, the parental strain maintained low H_2_ concentrations (Fig. 3B), as expected for a microbe that can consume H_2_ produced from Fe^0^ (17). These results indicated that H_2_ produced from Fe^0^ was an important electron donor for sulfate reduction by the parental strain.

However, the quantity of H_2_ that accumulated in abiotic Fe^0^-only controls or in the presence of Fe^0^ and the hydrogenase mutant was not sufficient to account for the amount of sulfate that the parental strain reduced with Fe^0^ as the electron donor. For example, on day 14 the parental strain had reduced 3.4 mM sulfate (50% of the time zero concentration of 6.8 mM), which would require 13.6 mM H_2_ (4:1 stoichiometry of H_2_ oxidized per sulfate reduced, reaction 2). Only about half that much H_2_ accumulated in the hydrogenase mutant cultures (Fig. 3B). One possibility for this disparity is that because rapid H_2_ uptake by the parental strain maintained low H_2_ concentrations (Fig. 3B), H_2_ production from Fe^0^ (reaction 1) was more thermodynamically favorable, possibly accelerating H_2_ generation over that in the hydrogenase mutant cultures in which H_2_ accumulated. We could not devise an experimental approach to abiotically mimic the expected rapid removal of H_2_ at the Fe^0^ surface. However, the alternative possibility that sulfide produced during the growth of the parental strain on Fe^0^ accelerated H_2_ production could be evaluated.

### Sulfide stimulates H_2_ production from Fe^0^.

**FIG 4:**
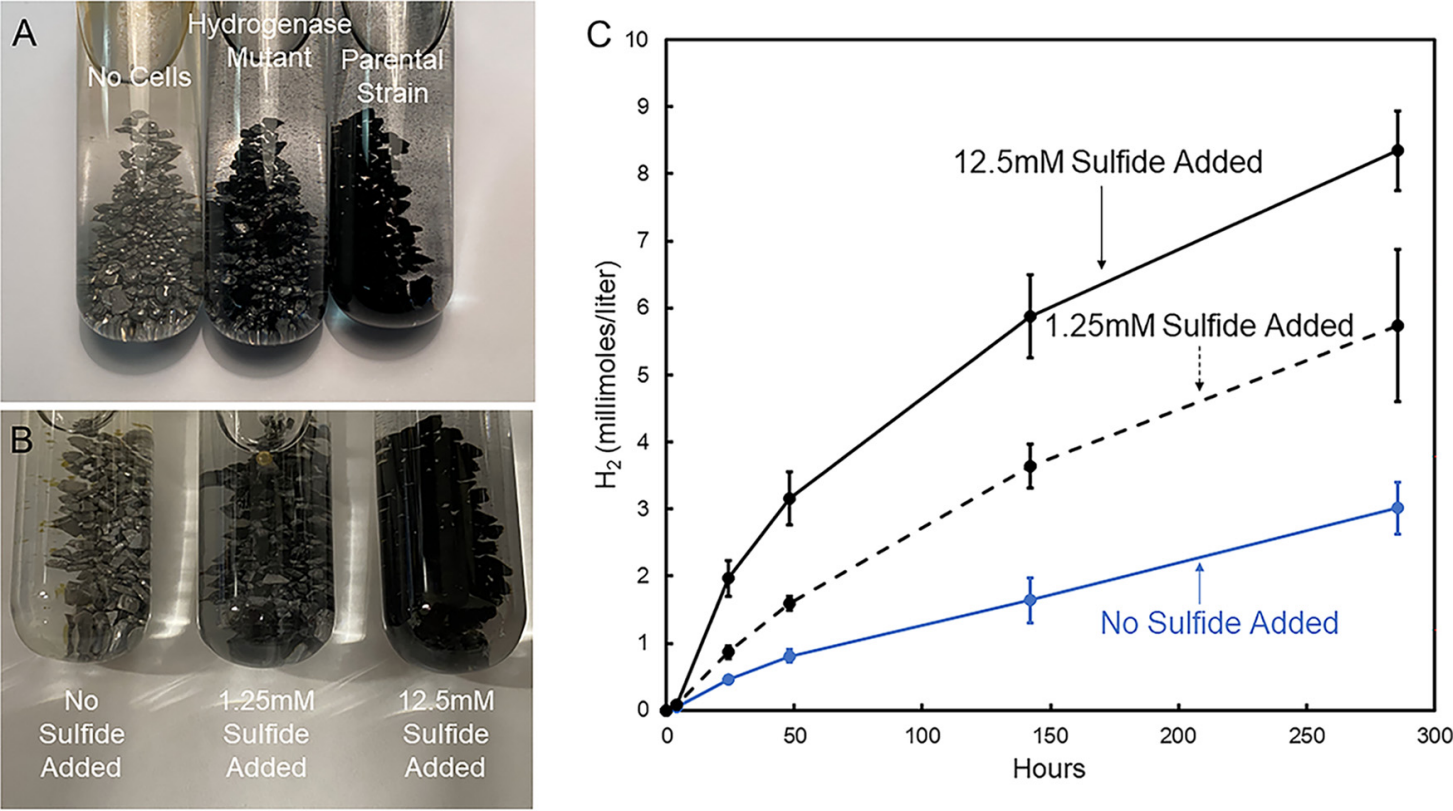
Iron sulfide accumulations in culture and impact of sulfide on abiotic H_2_ production. (A) Appearance of uninoculated Fe^0^-containing medium and cultures inoculated with either the hydrogenase mutant or parental strain after 14 days of incubation. (B) Appearance of sterile Fe^0^-containing medium amended with a 1.25 or 12.5 mM final concentration of sodium sulfide. (C) Accumulation of H_2_ over time in sterile Fe^0^-containing medium with and without added sulfide. The data represent the means and standard deviations of quadruplicate incubations.

Sulfide that the parental strain generated from sulfate reduction with Fe^0^ as the electron donor is also likely to have promoted H_2_ production (Fig. 4). Parental strain sulfide production was evident from the intense black precipitates indicative of iron sulfides on the Fe^0^ (Fig. 4A). In contrast, there was only a small amount of iron sulfide on the Fe^0^ of the hydrogenase mutant cultures, which could be attributed to sulfide transferred along with the inoculum (Fig. 4A). Sulfide was added to sterile medium, generating black iron sulfide precipitates (Fig. 4B), to assess the possible sulfide impact on H_2_ production. Adding sulfide stimulated H_2_ generation (Fig. 4C). One potential source of more H_2_ was the reaction of sulfide with Fe^0^ (reaction 3) in which there is a 1:1 stoichiometry for sulfide reacted and H_2_ produced. However, within 300 h the addition of 1.25 mM sulfide produced 2.7 mmol/L H_2_ (Fig. 4C), more than twice that expected from reaction 3. This result suggested that, as previously proposed (2, 10), iron sulfide precipitates also facilitated electron transfer from Fe^0^ to H^+^ (reaction 1), leading to additional H_2_ formation. The addition of 10-fold more sulfide only increased H_2_ an additional ~2-fold (Fig. 4C), further demonstrating a lack of defined stoichiometry between sulfide additions and H_2_ formation.

### Culture supernatants lack extracellular hydrogenase activity.

Hydrogenases released from some microbes can accelerate H^+^ reduction with Fe^0^ (30, 32, 33), and hydrogenase activity has been detected in supernatants of moribund *D. vulgaris* cultures (9). However, supernatants from *D. vulgaris* cultures grown either with H_2_ or Fe^0^ did not stimulate H_2_ production from Fe^0^ over that in abiotic controls (see Fig. S1).

10.1128/mbio.00076-23.1 FIG S1 H_2_ production from Fe^0^ in cell-free sterile medium or cell-free supernatant filtrate from parental strain D. vulgaris cultures grown with Fe^0^ or H_2_ as the sole electron donor. The results represent the means and standard deviations for triplicate incubations of each treatment. Download FIG S1, DOCX file, 0.1 MB. Copyright © 2023 Woodard et al. 2023 Woodard et al. https://creativecommons.org/licenses/by/4.0/ This content is distributed under the terms of the Creative Commons Attribution 4.0 International license.

### Stainless-steel studies confirm importance of H_2_ as intermediary electron carrier.

The inability of the hydrogenase mutant to reduce sulfate with Fe^0^ as the electron donor contrasts with electroactive microbes such as Geobacter sulfurreducens (17) or Shewanella oneidensis (23), which continue to utilize Fe^0^ as an electron donor even after gene deletions have eliminated the capability for H_2_ uptake. Both G. sulfurreducens and S. oneidensis are capable of direct electron uptake as evidenced from an inhibition of Fe^0^-based respiration when genes for key outer-surface *c*-type cytochromes are deleted (17, 23, 24). Thus, the lack of sulfate reduction by the *D. vulgaris* hydrogenase mutant when Fe^0^ was the electron donor suggests that it is incapable of direct electron uptake from Fe^0^.

This conclusion was further supported with the results of studies in which stainless steel was provided as the electron donor. Unlike pure Fe^0^, H_2_ production from stainless steel is minimal (18). However, microbes capable of direct electron uptake from Fe^0^ can extract electrons from stainless steel to support anaerobic respiration (18, 19, 24). *D. vulgaris* did not reduce sulfate with stainless steel as the electron donor (Fig. 3C).

### Electron shuttles do not promote Fe^0^-dependent sulfate reduction.

An alternative proposed electron transfer mechanism in Fe^0^ corrosion is that flavins shuttle electrons between Fe^0^ and *D. vulgaris* (Fig. 1A). An observed increase in Fe^0^ corrosion when riboflavin is added to *D. vulgaris* cultures has been offered as evidence for flavin shuttling (14, 28, 29). However, the riboflavin amendments were to complex medium in which lactate was provided as an electron donor in addition to Fe^0^. It was not demonstrated that the riboflavin additions increased rates of Fe^0^-dependent sulfate reduction. In order to examine the possibility of electron shuttles facilitating electron transfer between Fe^0^ and *D. vulgaris*, studies were conducted under defined conditions with Fe^0^ as the sole electron donor for sulfate reduction and either riboflavin or the known electron shuttle anthraquinone-2,6-disulfonate (AQDS) (34, 35). Riboflavin or AQDS did not accelerate Fe^0^-dependent sulfate reduction in the parental strain and did not enable the hydrogenase mutant to reduce sulfate with Fe^0^ as the electron donor (Fig. 3D). The midpoint potentials of AQDS (−184 mV) and riboflavin (−208 mV) are probably too positive for the reduced form of these molecules to support the reduction of sulfate to sulfide (midpoint potential, −217 mV). Therefore, the enhanced *D. vulgaris* Fe^0^ corrosion with riboflavin amendments (14, 28, 29) is likely to represent an impact of riboflavin on some aspect of growth or metabolism other than enhancement of electron transfer from Fe^0^ via an electron shuttle.

### *D. vulgaris* attaches to Fe^0^ electron donor.

**FIG 5:**
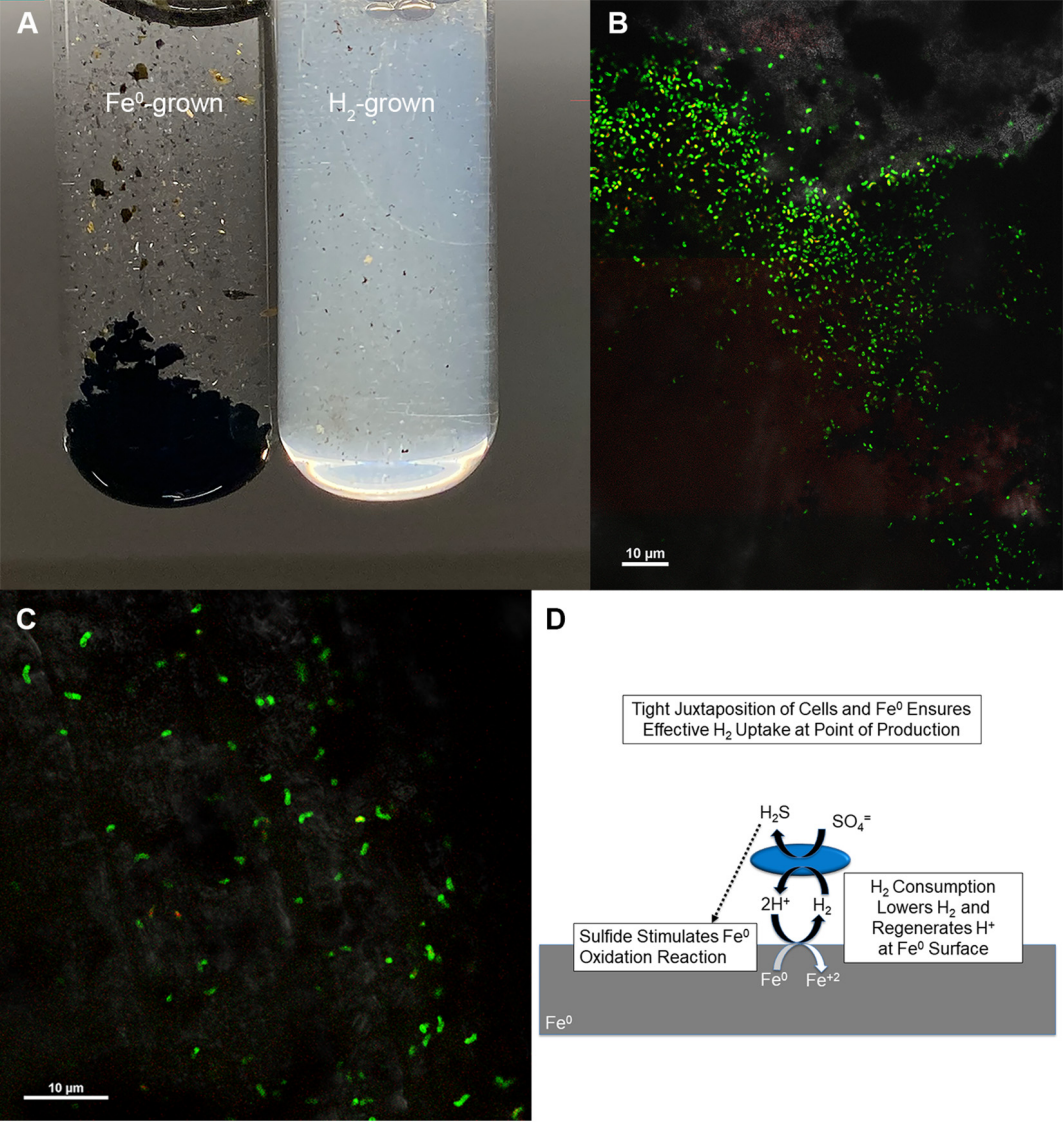
D. vulgaris parental strain attached to Fe^0^ serving as sole electron donor. (A) Lack of cell turbidity of culture growing with Fe^0^ as the source of H_2_ versus culture turbidity in culture grown under an H_2_ atmosphere. (B and C) Confocal scanning laser microscopy images showing green-stained cells scattered in plane with the dark metal background indicating that the cells are attached to the Fe^0^ surface. (D) Mechanisms by which attachment to Fe^0^ might accelerate H_2_ production.

The turbidity of *D. vulgaris* growing on Fe^0^ was very low compared to the turbidity in H_2_-grown cultures when a comparable amount of sulfate had been reduced (Fig. 5A). A portion of the cells in the *D. vulgaris* parental strain culture have a mutation that can, in the short-term (~100 h), delay attachment to glass surfaces (36), but confocal scanning laser microscopy revealed that cells colonized Fe^0^ (Fig. 5B and C). Individual cells were distributed across the Fe^0^ surface, without apparent cell stacking, in a manner similar to the surface growth of *Geobacter* (17), *Shewanella* (23), and *Methanosarcina* species (19) with Fe^0^ serving as the sole electron donor. The attachment of cells should be advantageous because it enables H_2_ uptake at the point of production where localized H_2_ concentrations are higher than in the bulk surrounding environment. Furthermore, localized conditions at the cell/Fe^0^ interface are likely to accelerate Fe^0^ oxidation (Fig. 5D). For example, attached *D. vulgaris* oxidizing H_2_ can make Fe^0^ oxidation more thermodynamically favorable, both by removing a product of the reaction (H_2_) and resupplying a reactant (H^+^) near the Fe^0^ surface. Sulfide produced at the Fe^0^ surface can further accelerate H_2_ production.

A common practice in Fe^0^ corrosion studies has been to infer that corrosion rates faster than that observed from abiotic H_2_ generation are indicative of corrosion mechanisms other than H_2_ serving as an intermediary electron carrier between Fe^0^ and cells (3). However, the possibilities for attached H_2_-consuming cells to accelerate H_2_ production from Fe^0^ illustrate the limitations to that reasoning.

### Implications.

Understanding how *D. vulgaris* promotes Fe^0^ oxidation is important because it is the microbe that has been used to develop much of the existing mechanistic framework to describe how sulfate reducers corrode Fe^0^ (7). The results demonstrate that the primary mechanism for *D. vulgaris* to reduce sulfate with Fe^0^ as an electron donor is with H_2_ serving as an electron shuttle between Fe^0^ and the cells. Sulfate was not reduced in the absence of genes required for H_2_ uptake, even when previously proposed organic electron shuttles were added. All the microbes that have been previously shown to be capable of direct electron uptake from Fe^0^ have outer-surface *c*-type cytochromes known to be involved in extracellular electron exchange with other donors/acceptors (17–19, 23, 24). *D. vulgaris* lacks outer-surface *c*-type cytochromes (25). Direct electron uptake from extracellular electron donors by routes other than cytochromes is possible (37). For example, several methanogen species that lack outer-surface *c*-type cytochromes appear to directly accept electrons from Geobacter metallireducens (38–41). However, the results presented here demonstrate that *D. vulgaris* does not function as an electrotroph with Fe^0^ as the electron donor. If *D. vulgaris* is representative of the sulfate reducers most responsible for the corrosion of ferrous metals, then potent hydrogenase inhibitors might provide a targeted approach to mitigate iron corrosion.

Microbes other than sulfate reducers also contribute to corrosion (3, 33, 42, 43). Elucidating the mechanisms by which a diversity of microbes accelerate corrosion is essential for understanding why corrosion takes place, predicting corrosion rates under various environmental conditions, and developing strategies for corrosion prevention. The studies reported here further demonstrate that construction of appropriate mutants is a powerful approach to distinguish between a complexity of potential corrosion mechanisms.

## MATERIALS AND METHODS

### Microbial strains.

Desulfovibrio vulgaris strains JW710 and JW5095, which were constructed in the laboratory of Judy Wall, University of Missouri (31, 44), were provided from a repository of *D. vulgaris* mutants by Valentine V. Trotter and Adam M. Deutschbauer of the Lawrence Berkeley Laboratory. Strain JW710 is a platform strain for a markerless genetic exchange system in *D. vulgaris* (44). The *upp* gene encoding uracil phosphoribosyltransferase has been deleted, to enable utilization of the *upp* gene as a counterselectable marker (44). Strain JW5095 was constructed by markerless deletion of all the hydrogenases that have been described in the *D. vulgaris* genome: DVU1921-22, DVU2525-26, DVU1917-18, DVU1769-70, DVU0429-34, DVU2286-93, and DVU1771 (31).

### Culture conditions.

Cultures were routinely grown anaerobically at 37°C in 10 mL of medium in 28-mL anaerobic pressure tubes (Bellco, Inc.) under N_2_/CO_2_ (80:20) in a modification of the previously described NBAF medium (45), designated NB medium. NB medium contains (per L of deionized water): 0.42 g of KH_2_PO_4_, 0.22 g of K_2_HPO_4_, 0.2 g of NH_4_Cl, 0.38 g of KCl, 0.36 g of NaCl, 0.04 g of CaCl_2_ · 2H_2_O, 0.1 g of MgSO_4_ · 7H_2_O, 1.8 g of NaHCO_3_, 0.5 g of Na_2_CO_3_, 1.0 mL of 1 mM Na_2_SeO_4_, 15.0 mL of a vitamin solution (46), and 10.0 mL of NB trace mineral solution. The composition of the NB trace mineral solution per L of deionized water is 2.14 g of nitrilotriacetic acid, 0.1 g of MnCl_2_ · 4H_2_O, 0.3 g of FeSO_4_ · 7H_2_O, 0.17 g of CoCl_2_ · 6H_2_O, 0.2 g of ZnSO_4_ · 7H_2_O, 0.03 g of CuCl_2_ · 2H_2_O, 0.005 g of AlK(SO_4_)_2_ · 12H_2_O, 0.005 g of H_3_BO_3_, 0.09 g of Na_2_MoO_4_, 0.11 g of NiSO_4_ · 6H_2_O, and 0.02 g of Na_2_WO_4_ · 2H_2_O. The medium pH was 6.7. Cells were routinely grown with sodium l-lactate as the electron donor (20 mM) and sodium sulfate (20 mM) as the electron acceptor. Growth was monitored by inserting culture tubes directly into a spectrophotometer and determining the *A*_600_ value. Growth with H_2_ as the sole electron donor was evaluated with 5 mM sodium acetate as a carbon source and H_2_ (140 kPa) as the sole electron donor. Cultures were incubated horizontally with shaking at 25 rpm and were routinely repressurized with H_2_ to compensate for any H_2_ consumption.

To evaluate growth with Fe^0^ as the potential electron donor, cells were grown in NB medium with Fe^0^ granules (2 g; 1 to 2 mm in diameter; Thermo Scientific) as the sole electron donor, 5 mM sulfate as the electron acceptor, and 5 mM sodium acetate as a carbon source. A 10% inoculum of a mid-log-phase culture of lactate-grown cells served as the inoculum. When specified, 50 μM riboflavin or 50 μM AQDS was added from concentrated anaerobic stock solutions. For studies with 316L stainless steel as the potential electron donor for sulfate reduction, five stainless steel cubes (5 mm × 3 mm × 3 mm) replaced the pure Fe^0^. The stainless-steel cubes were polished with sand paper, and the pure Fe^0^ and stainless steel were presterilized with ethanol as previously described (15).

### Impact of added sulfide or culture supernatant on H_2_ production.

A final concentration of either 1.25 or 12.5 mM sodium sulfide was added to sterile Fe^0^-containing medium to determine whether sulfide stimulated H_2_ production. Culture filtrates were prepared by filtering late-log-grown cultures (Fe^0^-grown or H_2_-grown) through a 0.2 μM PES filter in a Coy anaerobic glove bag (gas phase, 7:20:73 H_2_/CO_2_/N_2_) into pressure tubes with 2 g of Fe^0^. Tubes were resealed and flushed with N_2_/CO_2_ (80:20) for 5 min. Controls were sterile NB medium.

### Analytical methods.

For sulfate determinations, culture aliquots (0.1 mL) were anaerobically withdrawn with a syringe and needle, filtered (0.22 μm; polyvinylidene difluoride), and analyzed with a Dionex ICS-1000 with an AS22 column and AG22 guard with an eluent of 4.1 mM sodium carbonate and 1 mM sodium bicarbonate at 1.2 mL/min. H_2_ concentrations in the headspace were monitored on an Agilent 6890 gas chromatograph fitted with a thermal conductivity detector. The column was a Supelco Carboxen 1010 plot capillary column (30 m × 0.53 mm) with N_2_ carrier gas and 0.5-mL injections. The oven temperature was 40°C, the inlet was splitless at 5.5 lb/in^2^ and 225°C, and the detector had a makeup flow of 7 mL/min and a temperature of 225°C.

### Confocal microscopy.

For confocal microscopy, Fe^0^ was gently removed from the pressure tube, soaked in isotonic wash buffer for 10 min, drained, stained for 10 min (Live/Dead BacLight bacterial viability kit (Thermo Fisher); 1 mL staining with 3 μL of each stain per mL), and destained for 10 min in isotonic wash buffer. Fe^0^ pieces were then mounted on petri plates with an antifade/glycerol mixture. Cells were visualized with a 100× objective on a Nikon A1R-SIMe confocal microscope with NIS-Elements software.
