# Supplementary material for: H2 Is a Major Intermediate in Desulfovibrio vulgaris Corrosion of Iron
Source: mBio. 2023 Feb 14;14(2):e00076-23. doi: 10.1128/mbio.00076-23 (PMC10127678; doi:10.1128/mbio.00076-23)
Supplement: FIG S1 [file mbio.00076-23-s0001.docx]

**Supplementary Material**

**H_2_ is a Major Intermediate in *Desulfovibrio vulgaris* Corrosion of Iron**

Trevor L. Woodard^a^, Toshiyuki Ueki^a*^, and Derek R. Lovley^a,b#^

^a^Department of Microbiology, University of Massachusetts-Amherst, Amherst, MA, USA

^b^Institute for Applied Life Sciences, University of Massachusetts-Amherst

^*^Present address: Electrobiomaterials Institute, Key Laboratory for Anisotropy and Texture of Materials (Ministry of Education), Northeastern University, Shenyang 110819, China.

^#^Address correspondence to Derek R. Lovley, [dlovley@umass.edu](mailto:dlovley@umass.edu)


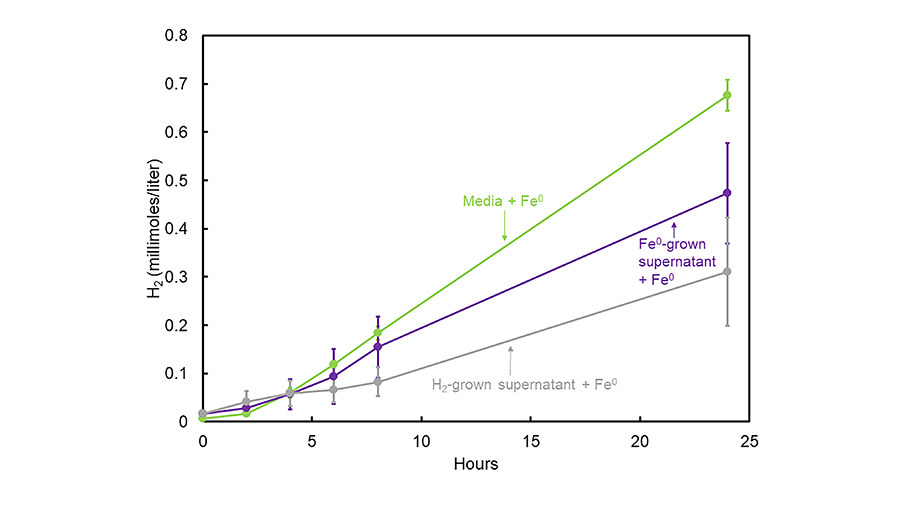


Supplementary Figure 1. H_2_ production from Fe^0^ in cell-free sterile medium or cell-free supernatant filtrate from parental strain *Desulfovibrio vulgaris* cultures grown with Fe^0^ or H_2_ as the sole electron donor. The results are the mean and standard deviation for triplicate incubations of each treatment.
